# Subclinical hypothyroidism in childhood, treatment or only follow-up?

**DOI:** 10.1186/s12887-020-02177-8

**Published:** 2020-06-06

**Authors:** Marta Murillo-Vallés, Santiago Martinez, Cristina Aguilar-Riera, Miguel Angel Garcia-Martin, Joan Bel-Comós, Maria Luisa Granada Ybern

**Affiliations:** 1 Endocrinology Unit. Pediatrics Section, Germans Trias i Pujol University Hospital, Autonomous University of Barcelona, Badalona, Spain; 2 Pediatrics Section, Germans Trias i Pujol University Hospital, Autonomous University of Barcelona, Badalona, Spain; 3 Clinical Biochemistry Department, Germans Trias i Pujol University Hospital, Autonomous University of Barcelona, Badalona, Spain

**Keywords:** Subclinical hypothyroidism, Cut-off, Child, Screening

## Abstract

**Background:**

Subclinical hypothyroidism (SH) is defined as serum levels of thyroid-stimulating hormone (TSH) above the upper limit with normal concentrations of free T4 (fT4). Its management remains challenging. The aim of the study was to evaluate clinical and laboratory findings as well as the clinical course of children with SH followed in a third level hospital. Sixty-five patients aged between 2 and 18 years old were retrospectively studied.

**Methods:**

The patients were followed for a median period of 9 months (range 6 months to 24 months). Those who normalized TSH levels were discharged (Group 1). If TSH persisted mildly elevated (5-10μUI/mL) with normal fT4 and negative TPOAb/TgAb, they were classified as Group 2 and followed semi-annually without treatment. Those patients whose TSH raised ≥10μUI/mL or who maintained TSH 5-10μUI/mL and positive TPOAb/TgAb were considered suitable for thyroxin therapy (Group 3, G3).

**Results:**

In 89% of our patients, TSH concentrations spontaneously reverted to normality or remained stable without treatment (Groups 1 and 2), whereas less than 11% progressed to clinical hypothyroidism (Group 3). Baseline TSH was significantly lower in group 1 than in group 3. In group 3 the prevalence of female sex (71%) was higher and TPO antibodies were present in 85% of patients. The risk of developing overt hypothyroidism in patients with positive anti-thyroid antibodies respect to those who normalized TSH was 45 (95%CI 6.5–312.5).

**Conclusion:**

Baseline TSH, female sex and the presence of thyroid autoimmunity were the best predictors of the evolution to SH over time.

## Background

Subclinical hypothyroidism (SH), also known as isolated hyperthyrotropinemia, is defined as serum thyroid-stimulating hormone (TSH) concentrations above the upper limit of the reference range and normal concentrations of free T4 (fT4). This situation occurs in less than 3% of children and adolescents [1, 2], but it is a cause of concern for parents and primary care physicians and represents a frequent cause of referral to a pediatric endocrinologist.

TSH normal range (0.4–0.5μUI/mL to 4.0–5.0μUI/mL) depends on the method used, with large variations found between different TSH assays. Idiopathic SH is characterized by mild elevations of TSH concentrations levels (5-10μUI/mL) with peripheral hormones fT4 and triiodothyronine within normal ranges, absence of thyroid autoimmunity or other conditions that may account for the increase in TSH, such as certain medications or genetic disorders (Down syndrome, Pseudohypoparathyroidism and others), and without clinical signs or symptoms of thyroid failure.

The natural course of idiopathic SH is unclear. Most patients normalize TSH values and a small percentage progresses to overt hypothyroidism [3–7]. The risk of progression to overt hypothyroidism depends on the cause of SH with high risk in autoimmune forms. There is a lack of conclusive studies that determine whether these children with SH might benefit from levothyroxine treatment [7–9].

On the other hand, adverse health outcomes of SH in childhood remain controversial. Although it might not produce adverse effects on growing and development processes [5, 8, 10], it has been recently associated with overweight/obesity and metabolic abnormalities [11, 12]. Nevertheless, prospective studies that determine those deleterious effects are lacking.

This study aimed to analyze the characteristics and natural evolution of a cohort of children with SH referred to a third level hospital.

## Methods

We analyzed retrospectively patients who were diagnosed with SH and referred to the Endocrinology Unit of our hospital between 2014 and 2018. SH was defined as TSH concentration mildly elevated (5-10μUI/mL) with fT4 within the normal reference range (0.7–1.48 ng/dL). Patients were assessed at the time of diagnosis, at month 3 and every 6 months during follow-up if necessary.

### Patients

Inclusion criteria were as follows: patients referred for SH aged 2 to 18 years old with at least two analytical records: one at the time of diagnosis and another one during the follow-up. Patients under 2-years-old and who received pharmacological treatment that could alter the TSH concentrations (anticonvulsants, antipsychotics, glucocorticoids, iodine or iodine-rich diet) were excluded. We also excluded patients with genetic syndromes or under an acute disease. All patients resided in an area by the Mediterranean Sea in an iodine-sufficient population.

All patients had a complete clinical record, physical examination including anthropometric characteristics (height, weight), and thyroid exploration at the time of diagnosis and during follow-up visits. We calculated body mass index (BMI) and represented the results as standard deviation (SD) according to age and sex. Obesity was considered if the BMI-SD was above 2 for the reference population. Short stature was considered if the height-SD was below 2 for the reference population.

Thyroid function test consisted of TSH, fT4 and thyroid autoantibodies (anti-peroxidase (TPOAb) and antithyroglobulin (TgAb)). If necessary –p.e. with palpable goiter, persisted elevated TSH or positive autoimmunity–, a thyroid ultrasound was made to assess thyroid size and echogenicity.

All patients had an initial TSH concentration mildly elevated (5-10μUI/mL) with fT4 within the normal reference range. The patients were followed for a median period of 9 months (range 6–24 months). Those patients who normalized TSH levels at the follow-up were discharged (Group 1, G1); follow-up median period 6 months (range 3–9 months) whereas those who persisted with elevated TSH were followed. If TSH persisted mildly elevated (5-10μUI/mL) with normal fT4 and negative TPOAb/TgAb, they were classified as Group 2 (G2) and followed semi-annually without treatment, follow-up median period 12 months (range 9–24 months). Those patients whose TSH raised ≥10μUI/mL or who maintained TSH 5-10μUI/mL and positive TPOAb/TgAb (regardless fT4 levels) were considered overt hypothyroidism and suitable for thyroxin replacement therapy due to hypothyroidism/thyroiditis (Group 3, G3), follow-up median period 12 months (range 9–24 months).

### Biochemical and hormonal determinations

Blood samples were drawn at 8 a.m. after an overnight fast. Samples were centrifuged and sera kept frozen at − 20 °C until analysis. Analysis of serum TSH was performed with CLIA with the aid of an Abbott ARCHITECT instrument (Abbott Diagnostics Division). Total coefficient of variation (CV) was < 3.3%, functional sensitivity was 0.0038μUI/mL; reference range [99% confidence interval (CI)]: 0.35–4.94μUI/mL; fT4 was measured by Abbott ARCHITECT instrument (Abbott Diagnostics Division; total CV was < 7%, sensitivity of the assay was < 0.4 ng/dL; reference range (99% CI): 0.70–1.48 ng/dL (conversion factor ng/dL *12.87 = pmol/L). TPOAb were measured by an Abbott ARCHITECT instrument (Abbott Diagnostics Division). Total CV was < 7.6%, sensitivity of the assay was 0.16 IU/mL; reference range: < 5.61 IU/mL.

### Statistical analyses

The normality of the evaluated variables was established using the Kolmogorov-Smirnov test. Quantitative variables are presented as mean ± SD or as median (interquartile range: 25th–75th percentile) whereas qualitative variables are expressed as percentages. Differences among groups were assessed by the ANOVA or the non-parametric Kruskal Wallis test. To determine differences between groups, the Student’s T test or non-parametric Mann-Whitney U test was used, and to compare variables at baseline and at follow-up, we used the Wilcoxon test. Differences in proportions were analyzed by the χ^2^ test or Ficher’s exact test. All tests were two-tailed and a p value < 0.05 was considered statistically significant. Statistical analyses were performed using the SPSS package 12.0 and MedCalc Software 12.7.0 (Acacialaan 22, B-8400 Ostend, Belgium).

The relative risk was calculated as the ratio of the proportions of cases having a positive antibodies in the G3 group (6 out of 7), respect to group G1 (0 out of 44). This test was performed with the aid of MedCalc Statistical Software version 19.1.7 (MedCalc Software Ltd., Ostend, Belgium; https://www.medcalc.org; 2020) The program calculates the relative risk with 95% confidence interval, the z-statistic and associated P-value. If P is less than 0.05, it can be concluded that the proportions are significantly different in the two groups, and there is an increased risk in one group compared to the other.

The study was approved by the Ethics Committee. All patients or legal surrogates gave informed consent prior to participation.

## Results

The study included 65 patients diagnosed with SH (51% female). The median age at diagnosis was 7.75 (4.4–9.17) years old. Clinical and laboratory findings at the time of diagnosis are shown in Table 1. The main reason for the study of thyroid function was routine analytical (41,5%, n: 27), obesity (13,8%, n: 9), short stature (12.3%, n: 8), asthenia (10.7%, n: 7) or others (18.4%, n: 14).

**Table 1:** Anthropometrical, clinical and laboratory characteristics at baseline

n: 65	
Female (%)	33 (51%)
Age (years)	7.75 (4.4–9.17)
Referral for analytic study:	
Routine	27 (41.5%)
Obesity	9 (13.8%)
Short stature	8 (12.3%)
Asthenia	7 (10.7%)
Others	14 (21,5%)
Clinical features:	
BMI-SD	−0.5 [(−1.09)-(+ 0.86)]
Weight-SD	−0.39 [− 0.99)-(+ 1.2)]
Height-SD	− 0.47 [(− 1.38)-(+ 0.75)]
Obesity (BMI-SD ≥2)	9 (13.8%)
Short stature (<2SD)	8 (12.3%)
Goiter	0
Familiar history (Autoimmune disease)	8 (12.3%)
Laboratory results:	
TSH (μUI/mL)	6.7 (6.1–7.8)
Free thyroxin (ng/dL)	1.1 (1.0–1.3)
TPO positivity	6 (9.2%)
US performed (%)	23 (35,3%)

Data are expressed as median (IQR) or percentage (%)

Regarding clinical characteristics, the medians of weight, height and BMI were within the normal ranges (− 0.39SD, − 0.47SD and − 0.5SD respectively) with 13.8% of obesity and 12,3% of patients with short stature. No patient had goiter on physical examination.

There was a family history of thyroid disease in 8 cases (12.3%).

At the time of diagnosis, all patients were asymptomatic of thyroid dysfunction, and these patients were observed without treatment.

Table 2 shows the clinical and laboratory characteristics of patients classified into 3 different groups according to evolution. TSH concentrations returned to normal ranges in 44 patients (67.6%) (G1), 14 patients (21.5%) maintained slightly elevated TSH concentrations with negative thyroid antibodies (G2) and 7 patients (10.7%) had TSH ≥10μUI/mL or TSH 5-10μUI/mL and positive TPOAb/TgAb (6 patients) (G3).

**Table 2:** Clinical and laboratory characteristics among groups

	Group 1	Group 2	Group 3	Significance
N	44 (67,6%)	14 (21,5%)	7 (10,7%)	
Female	51.2%	42.9%	71.4%	N.S.
Age at baseline (years)	6.7 (4.0–9.2)	8.17 (6.48–9.48)	8.17 (7.4–9.17)	N.S.
Familiar History (Autoimmune disease)	11.4%	7.1%	14.3%	N.S.
TSH (μUI/mL) at baseline	6.6 (6.02–7.35)^a^	7.05 (6.07–8.12)	8.2 (6.7–9.4)	0.012
Free thyroxin (ng/dL) at baseline	1.15(1.02–1.3)	1.0(0.95–1.3)	1.0(0.88–1.1)	0.03
TSH (μUI/mL) at follow-up	3.7(2.94–4.27^)b, c^	6.1(5.17–8.0)^d^	1.0(088–1.1)	< 0.001
Free thyroxin (ng/dL) at follow-up	1.07 (0.98–1.2)	1.04(0.9–1.19)	0.88 (0.83–1.1)	N.S.
Percentage change in TSH (baseline vs follow up)	−47.8 ((−35.3)-(− 56.7))^b,c^	−6.9 ((−17.3)-1.9)^d^	24.7 (14.6–95.5)	< 0.001
BMI-SD	−0.61 [(−1.1)-0.83]	0.21 [(− 0.62)-1.18]	0.43[(− 0.68) − 0.85]	NS
Obesity (BMI ≥2 SD)	6 (13,6%)	3 (21,4%)	0 (0%)	N.S.
Height-SD	-0.85 [(− 0.98)-0.67]	−0.45 [(2.27)-0.82]	0.35 [(−2.2)-1.4]	N.S.
Height-SD <2SD	1 (2,2%)	5 (35,7%)	2 (28,5%)	N.S.
TPOAb/TgAb positivity	0 (0%)	0 (0%)	6 (85%)	0.001
Performed US	10 (22%)	6 (42%)	7 (100%)	
Tiroiditis by US	0 (0%)	1 (16%)	6 (83%)	0.04

N.S.: non significant

^a^: p < 0.016group 1 vs group 3

^b^: p < 0.001 group 1 vs group 2

^c^ p < 0.001 group 1 vs group 3

^d^: p < 0.001 group 2 vs group 3

Quantitative variables are expressed as median (IQ range p25-p75)

Qualitative variables are expressed as N (%)

The prevalence of females was higher in the G3 (71%), but in the other two groups, the distribution was similar (50% in G1 and 57% G2). Regarding the age as well as a family history of autoimmune diseases, no differences were found among the 3 groups.

**Fig. 1:**
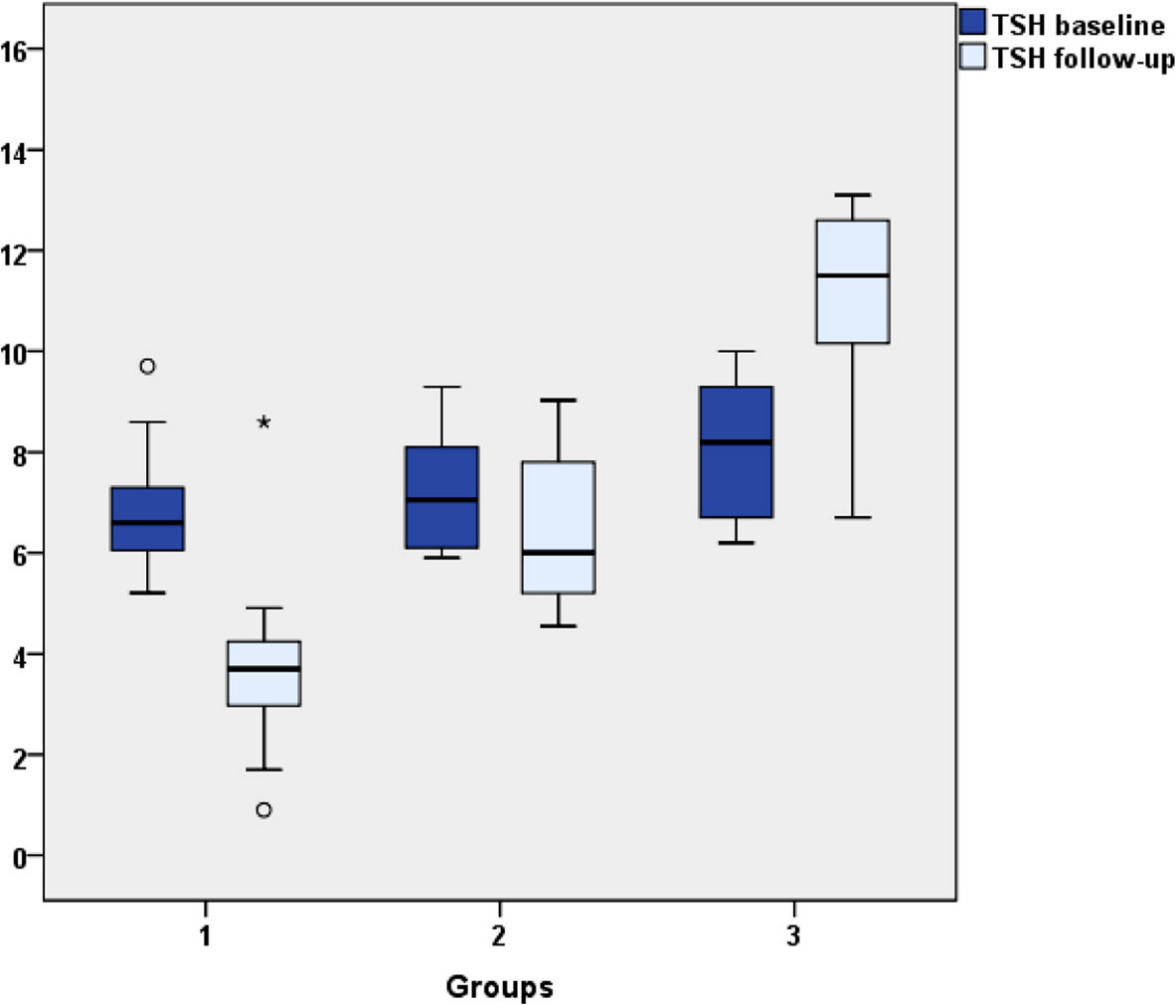
TSH values at baseline and follow-up. Patients were classified into three groups according TSH levels at the end of the follow up. Group 1 (G1): TSH ≤5μUI/mL (n: 44); Group 2 (G2): TSH 5-10μUI/mL, normal fT4 and negative TPOAb/TgAb (n: 14); Group 3 (G3): suitable for thyroxin therapy due to TSH ≥10μUI/mL or TSH 5-10μUI/mL and positive TPOAb/TgAb (n: 7)

Mean baseline TSH concentrations differed among the 3 groups (p = 0.012), significant differences were found in TSH values between G1 and G3 (F = 4.768; p = 0.016) whereas no differences were found between G1 and G2 or between G2 and G3. No differences were found in fT4 concentrations at baseline among the 3 groups (F = 3.083; p = 0.053) (Fig. 1). The percentage change of TSH at follow-up with respect to baseline was 47.8% decrease in group 1, a 6.9% decrease in group 2 and a 24.7% increase in group 3 (Table 2).

BMI evolution was evaluated during the study and no significant difference was found in obesity (BMI-SD > 2) prevalence during follow-up in all patients (13,8 to 10.7%). In terms of height there were no differences.

Thyroid autoimmunity was observed in 85% of patients of G3 and none of G1 and G2 (p > 0.001). The risk of developing overt hypothyroidism in patients with positive anti-thyroid antibodies with respect to those who normalized TSH was 45 (95%CI 6.5–312.5).

Of all the patients, 35,3% underwent a thyroid ultrasound and a structure suggestive of thyroiditis (heterogeneous, hypoechoic and in some cases enlarged ultrasound pattern) was found in 6 patients, all in the G3. There was 1 patient with nodules on ultrasound without other significant findings; the rest of the patients presented a normal ultrasound.

## Discussion

In general terms, SH seems to affect less than 3% of the child population and usually displays a natural course towards the maintenance or spontaneous resolution in variable time in most cases (68–88%) and only a few cases progress to overt hypothyroidism or autoimmune thyroiditis. There are few studies to date that evaluate SH and its evolution in childhood [5, 10, 13, 14]. In our study, most of the patients either normalized their TSH levels or maintained their TSH levels under subclinical range values (89%). Only a small percentage presented overt hypothyroidism or autoimmune thyroiditis and needed treatment (11%). These data are similar to those of a 2-year prospective study presented by Wasniewska et al., in which 88% of patients normalized o preserved their TSH levels and 12% developed hypothyroidism [5].

In an attempt to anticipate events, Lazar et al. found that predictive factors for sustained abnormal TSH levels were initial TSH > 7.5μUI/mL and female gender [1]. Recently, Gammons et al. concluded that a TSH >8μUI/mL would be the cut-off point to refer to a pediatric endocrinologist for evaluation and management [15]. Although, because of the small number of patients, an AUC could not be assessed to determine the cutoff level of TSH that predicts evolution towards hypothyroidism, in our cohort baseline TSH levels of G3 were significantly higher.

Regarding the weight, in our study, no significant changes were detected between obesity prevalence at baseline and during follow-up in all the patients, and the BMI-SD did not worsen during follow-up in any patient. However, in the G2 group, those who persisted with a TSH 5-10μUI/mL had a higher prevalence of obesity, which would suggest that it may have a role in increasing TSH levels. It is thought that the mildly elevated TSH is the consequence of obesity rather than the cause, as an attempt to increase energy expenditure, with an improvement of the thyroid function parameters when lowering the BMI, as has been mentioned on multiples studies [16–21]. Contrary to what one would expect, none of the patients with obesity belonged to the G3 with overt hypothyroidism.

It is known that autoimmunity is a key factor that determines the major progression to hypothyroidism. In our study, almost all patients in G3 met this condition and 71.4% were women, which is a distribution that already occurs in most autoimmune diseases. Our results are in agreement with Wasnieska et al. [22] who studied the long term evolution of a large cohort of girls with subclinical hypothyroidism and found that underlying Hashimoto thyroiditis was the main factor to become overtly hypothyroid or require L-T4 treatment. In fact, it has been reported that in children with Hashimoto thyroiditis the evolution of thyroid status is frequently characterized by a spontaneous worsening over time, even in the cases who initially present with a mild biochemical picture [23]. Lazarus et al. analyzed the results of 7 observational studies and showed that elevated TgAb and TPOAb at diagnosis were associated with an increased risk of progression in some but not all studies [7].

Treatment with levothyroxine was initiated in all patients who presented TSH ≥10μUI/mL or TSH 5-10μUI/mL and positive TPOAb/TgAb (G3). The dilemma arises in deciding whether patients with maintained mildly elevated TSH (G2) should be treated or not with levothyroxine and what benefits it can bring against possible consequences of SH, since good-quality studies examining the effect of treatment of SH in children are lacking [9, 24–26]. In our case, no patients were treated since none presented associated symptoms or alterations that could be related to it.

The current study has its limitations. For instance, it is a retrospective study with a small number of patients, and genetic causes such as alterations in the TSH receptor that could explain mildly elevated TSH have not been investigated. On the other hand, the possible impact on growth and intellectual development has not been assessed in this study.

## Conclusion

Although SH in childhood is a frequent issue and a matter of concern between primary care pediatrics, it seems to be a benign and remitting condition; based on our results and in comparison, with the literature, expectant behavior is the best option, always individualizing each patient. Perhaps repeating a second determination by the primary care pediatrician of TSH and fT4 in 1–3 months and if alteration persists refer to specialist many cases could be resolved and, therefore, save time and resources.

However, in the case of pediatric SH, prospective studies are lacking to determine a sensitive and specific level of TSH to predict the progression to hypothyroidism. It is important to determine if it is a process with negative or positive autoimmunity, since on this latter case, the probability of progression to hypothyroidism is greater. As seen in the current study, baseline TSH, female sex and the presence of thyroid autoimmunity were the best predictors of the evolution to SH over time.

## Data Availability

The datasets during and/or analyzed during the current study are available from the corresponding author on reasonable request.

## Abbreviations

TPOAb: Anti-peroxidase thyroid autoantibody
TgAb: Antithyroglobulin thyroid autoantibody
AUC: Area Under Curve
BMI: Body mass index
CI: Confidence interval
fT4: Free T4
G1: Group 1
G2: Group 2
G3: Group 3
ROC: Receiver Operating Characteristic
SD: Standard deviation
SH: Subclinical hypothyroidism
TSH: Thyroid-stimulating hormone
CV: Total coefficient of variation
